# Extensive vegetation browning and drying in forests of India’s Tiger Reserves

**DOI:** 10.1038/s41598-019-51118-8

**Published:** 2019-10-18

**Authors:** Pradeep S. Koulgi, Nicholas Clinton, Krithi K. Karanth

**Affiliations:** 1 Centre for Wildlife Studies, 37/5, Yellappa Chetty Layout, Ulsoor Road (Off Halasuru Road), Bengaluru, 560042 India; 2 Google, Inc., 1600 Amphitheatre Pkwy, Mountain View, CA 94043 USA; 3 Duke University, Durham, North Carolina USA

**Keywords:** Conservation biology, Forest ecology

## Abstract

Forest conservation includes stemming deforestation as well as preserving its vegetation condition. Traditional Protected Area (PA) effectiveness evaluations have assessed changes in forest extent but have mostly ignored vegetation condition. Tiger Reserves (TRs) are India’s PAs with highest protection and management resources. We used a before-after-control-impact-style design with long-term Landsat 5 TM data to evaluate the effects of protection elevation on vegetation condition (greenness and moisture) in 25 TRs. After declaration as TRs, vegetation condition in 13 TRs (52%) declined in more than 50% of their areas, with 12 TRs (48%) being overall better than their matched Wildlife Sanctuaries (WLSs; PAs with lower protection). In 8 of these TRs analysed for change from before to after declaration, vegetation condition in 5 TRs was harmed over more than 25% of their areas, with 3 TRs being overall better than their matched WLSs. Our results indicate extensive vegetation browning and drying in about half of the study TRs, with these trends often being similar or worse than in matched WLSs. These results suggest that TRs’ elevated protection alone may be insufficient to preserve vegetation condition and cast doubt on the effectiveness of protection elevation alone in safeguarding long-term viability of tiger habitats.

## Introduction

Terrestrial Protected Areas (PAs) cover roughly 15% of land globally^1^ and help protect a majority of the threatened taxa, making them central to ongoing and future biodiversity conservation efforts globally^2^. Goals of PA establishment and management often reflect local, regional, and national conservation priorities, leading to different protection levels for their forests and biodiversity^3,4^. *In*-*situ* conservation of single species such as umbrella or flagship species^5–7^ is one such goal with profound implications to on-ground PA protection and management practices^8,9^. Effectiveness assessments of PAs and their focal species-oriented management in achieving their conservation goals are essential for planning and adaptive management as well as in the allocation of scarce conservation resources^10,11^. However, direct assessments of progress towards biodiversity and habitat conservation goals are rare^12^ and rendered difficult by data inadequacies, with previous effectiveness evaluations often adopting a generic PA management-oriented approach^13^.

Effective forest conservation includes stemming deforestation as well as preserving condition of its habitat and vegetation. Previous PA evaluations have mostly assessed deforestation (i.e., forest cover loss, habitat loss), and have found considerable variation in their effectiveness in halting it: eg., PAs across the tropics have often seen reductions of deforestation within them^14,15^, but not without notable exceptions (see examples in south-east Asia and China^16,17^). Across a gradient of protection and management resource availability, PAs with lower protection and resources can be less effective in averting deforestation^18^. Assessment of forest vegetation condition, on the other hand, has hardly figured in such evaluation frameworks. This omission assumes further importance when PA protection level and management align with conservation prioritization such as umbrella species conservation schemes, which promise intended indirect conservation benefits to forest condition and ecological processes^19,20^. Given the strong influence of such prioritization on ground-level PA management practices^8^ and the growing recognition of the importance of forest vegetation condition changes as targets and triggers for forest management^21–23^, it is crucial to explicitly include vegetation condition assessments in PA effectiveness evaluations.

Reflecting trends elsewhere in the world, India adopted a formal PA-driven approach to conserving its forests and biodiversity in the 1970s. Additionally, India started Project Tiger in 1973 to bring an umbrella species approach to *in*-*situ* conservation of the tiger (*Panthera tigris*) and its forest habitat, and is presently the wild tiger’s last stronghold globally^24^. Effectiveness evaluations of Indian PAs are in their infancy^25,26^ and are yet to include vegetation condition assessments in their evaluation framework. Forest protection and management regimes in India range from strict protection for biodiversity conservation to those allowing extractive use and joint community management, with Project Tiger-designated Tiger Reserves (TRs) being the country’s most strictly protected PAs and with greatest management resources outlay^27,28^. The government has undertaken TR evaluations a few times since mid-2000s^26,29,30^, but they follow a generic management-centric approach and rely heavily on expert assessments (also^31^). The India State of Forest Report prepared periodically by Forest Survey of India, a nodal agency under the federal government tasked with surveying and assessing forest resources in India, describes detailed forest cover extent assessments (see^32^ for a critique) using data from multiple modalities but fails to feature vegetation condition assessments. Population estimates from periodic nation-wide tiger census undertaken by government agencies show an increasing trend at the national level, and these are used in government reports claiming conservation successes, but methodological inconsistencies between censuses and use of disputed census methods^33,34^ have rendered these estimates unreliable for scientific evaluations. Recent attempts at evaluating forest change have incorporated data from multiple modalities (eg. on-ground, remotely sensed, management parameters) but are restricted to a single PA or a small local cluster of them^35,36^. Recent emergence of time series satellite data analysis for forest assessment has opened up more powerful ways of assessing forested landscapes^37–39^, including in TRs and critical tiger habitats at sub-continental scale^40^. However, there is yet to be a published scientific effectiveness evaluation of protection elevation of PAs, and umbrella species conservation scheme, in conserving forest habitat across India that explicitly incorporates forest vegetation condition.

In this study, we evaluated the effectiveness of TRs’ maximum protection status in conserving their vegetation condition, using a study design based on before-after control-impact (BACI) paired design^41^ which is well suited for detecting and quantifying the effects of human interventions on natural systems^42^. We matched TRs (“impact”s) with PAs having lower protection status (“control”s) (Wildlife Sanctuaries (WLSs); see Methods) and used the roughly 3 decades long (1984–2012) Landsat 5 TM satellite time series data on the Google Earth Engine cloud computing platform^43^ for before-after analyses of vegetation condition with respect to TR declaration. From remote-sensed estimates of vegetation greenness (enhanced vegetation index (EVI)) and moisture content (normalized difference infrared index (NDII)), we estimated trends in temporal change (slopes of linear regression) of annual vegetation condition across these PAs in the epochs “before” and “after” TR declaration. First, we performed a matched control (WLS) - impact (TR) comparison of estimated trends in vegetation condition changes in the years after TR declaration. Second, in order to account for historical context in these trends, we performed a matched control-impact comparison of change in these trends (difference of slopes) from before to after TR declaration. We hypothesized that the declaration of TRs will benefit the forest vegetation condition, for two reasons: first, reduced degradation of forest vegetation and facilitation of its recovery due to greater investments in ground-level patrolling and higher protections against human-use; and second, expected indirect vegetation and habitat conservation benefits from umbrella species-oriented management of TRs to conserve tiger as its umbrella species. We predicted the TRs to show more of its forest vegetation improving in the years after, and being helped by, TR declaration, compared to their matched WLSs.

## Methods

### Study area

The Indian terrestrial PA network covers about 5% of its land area and is comprised of over 600 PAs with National Parks (NPs), Wildlife Sanctuaries (WLSs), Conservation Reserves (CNRs) and Community Reserves (CMRs) spanning a range of protection levels^27^. WLSs and NPs are managed solely by the government with NPs receiving greater protection than WLSs, while CNRs and CMRs can be multiple-use forests managed by the government jointly with local communities. Some WLSs, NPs, or a combination of them, that constitute important habitats for *in*-*situ* conservation of the tiger are elevated to Tiger Reserve (TR) status and are brought under the purview of Project Tiger – Indian federal government’s most prestigious conservation initiative. Consequently, TRs receive the maximum legal protection of all PAs as well as special extra funding through federally sponsored schemes making their budgetary outlay significantly greater than other PAs in the country^28^. Project Tiger began over four decades ago in 1973, with the aim to conserve the tiger in its habitat as an umbrella and flagship species. Beginning with declaring 9 TRs at the start of the project, there are now 50 TRs with about a third of them declared in the last ten years. Today’s TRs are distributed across 18 states, grouped under 5 management clusters^26^, range in size from roughly 140 *km*^2^ to 2600 *km*^2^ and together cover about 71000 *km*^2^. They represent the diversity of habitats the tiger is found in, encompassing India’s subtropical evergreen and moist deciduous forests of the Western Ghats and North Eastern hills, dry deciduous and scrub forests of Central Indian highlands, deciduous forests of Western and Central Himalayan foothills as well as mangroves of the Gangetic delta. While all PAs, including TRs, fall under the purview of the federal government’s Ministry of Environment Forests and Climate Change, each PA is administered and managed at the state level.

### Data

#### Remotely sensed data

The seven satellites of the United States’ Landsat mission to-date, together spanning over four decades, provide the longest remotely sensed continuous land data record of the earth^44^. Technical challenges remain for time-series analyses that require harmonizing data from multiple Landsat sensors. We selected Landsat 5 TM data for being the single longest running satellite of the family with a land data record spanning the longest duration (nearly 29 years, from 1984 to 2012). We used its Collection-1 Tier-1 atmospherically corrected surface reflectance product, due to its suitability for time-series analysis^45^. For estimates of landscape-scale long-term average annual rainfall to match PAs by similar forest types, we used gridded monthly average precipitation data from the WorldClim Climatology V1 dataset^46^.

#### Indian Protected Areas

Our dataset of mainland India’s terrestrial PAs included administrative boundaries of 473 PAs and their legal protection status (WLS, NP or TR) as of the year 2012 (end of Landsat 5 TM data and, hence, our study period). This dataset was collated from prior published studies^47^, government agencies^48^, diverse civil society organizations and field personnel, and verified against government notifications of PA declarations where possible (Nayak *et al*., in review; Karanth *et al*. unpublished). PAs predominantly containing water bodies such as rivers or lakes and constituted for conserving riverine or freshwater species and habitats were excluded from our analysis.

**Figure 1:**
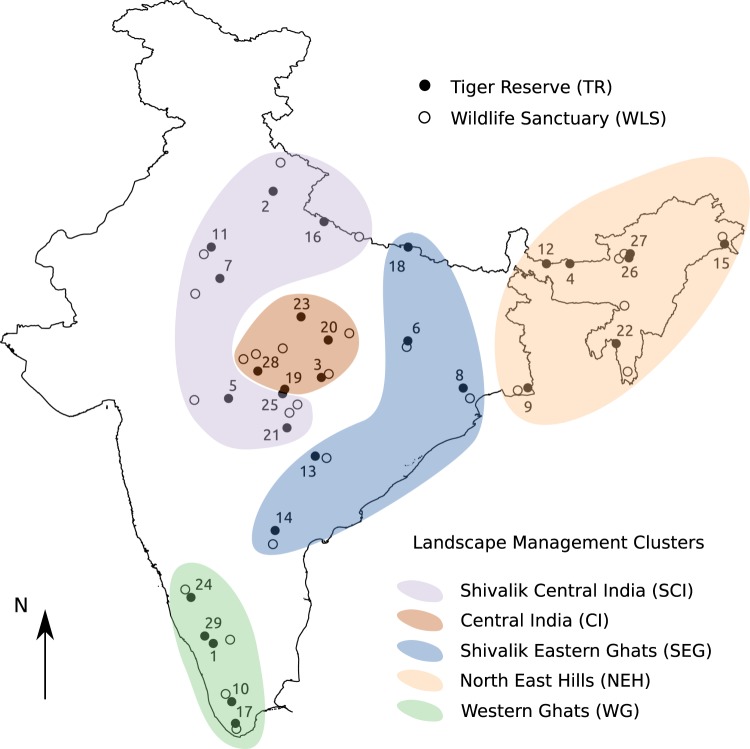
Map of India showing locations of PAs in our study: the 29 Tiger Reserves (TRs, ‘●’) declared in the year 2000 or earlier and numbered in chronological order of their year of declaration, and their matched Wildlife Sanctuaries (WLSs, ‘○’). The 25 matched TR-WLS pairs with adequate data for analysis are in Table 1. Enclosing colored patches denote the landscape management clusters the TRs are included in.

Of the 50 currently existing TRs, we selected 29 that were declared in the year 2000 or earlier, which span all five management clusters: Central India (CI), North East Hills (NEH), Shivalik - Central India (SCI), Shivalik - Eastern Ghats (SEG) and Western Ghats (WG) (Fig. 1). With our study period spanning 1984–2012, this set of TRs allows a decade-long time series for estimation of trends post-declaration for all of them, and both pre- and post-declaration time series for a subset of them. Subsequent TR declarations were not until 2008, coming after 2006 when landmark new amendments (to the Wildlife (Protection) Act of 1972) and legislation (Scheduled Tribes and Other Traditional Forest Dwellers (Recognition of Forest Rights) Act, 2006) were passed impacting forest governance nation-wide. However, these legislative changes, being close to the end of our study period, are unlikely to impact our measurements and hence can be ignored for the purposes of our study. For the purpose of this study the year of declaration of WLSs in our dataset was ignored and all the selected ones were not elevated in protection till the end of our study period (year 2012). Additionally, here we treat PAs in the state of Telangana as being in Andhra Pradesh (AP), since Telangana got carved out of AP and acquired separate statehood after 2012.

#### Matching Tiger Reserve – Wildlife Sanctuary pairs

We matched each TR with a WLS of area 75 *km*^2^ or greater, having a similar forest-type and shared governmental management framework. We controlled for governmental management and administrative variations by restricting matched pairs to lie within the same state, given that on-ground management of PAs in India is at the state-level. Using long-term average annual rainfall to control for forest vegetation type, for each TR we selected a subset of WLSs within the same state and having an average annual rainfall within 500 *mm* from the TR’s. From this subset we assigned the TR its matched WLS as the one at the shortest distance from the TR. In case of a tie between two TRs, we chose a tie-breaking WLS for one of them as the next nearest WLS from within this subset.

A crucial role of the matched WLSs in our study derives from them being PAs with lower protection than TRs in India. To ensure that this within-matched-pair difference in protection levels was retained throughout our study period and the comparison remained valid, we ensured that the chosen WLSs were not elevated in protection within our study period and ignored their year of declaration. If they were declared as WLS within the study period, the comparison still remains valid because the within-matched-pair difference in protection levels still remains and more amplified, since a WLS would have lesser protection prior to its declaration as a WLS.

#### Vegetation condition annual composites

Forest vegetation browning and greening, as measured by vegetation indices using satellite imagery, have been used to quantify forest ecosystem properties^49–51^. Preserving them and facilitating their recovery are critical aspects of forest conservation. In this study we investigate trends in both vegetation greenness and moisture, two complementary remotely sensed metrics of vegetation condition^52^, to assess forest vegetation condition. Simultaneous gains in both metrics indicates condition improvement in an epoch, or condition being helped by an intervention from its before to after epochs. Similarly, simultaneous diminishment in both metrics indicates condition decline in an epoch, or condition being harmed by an intervention from its before to after epochs. The condition is unclear when there are gains in one metric and diminishment in the other.

**Figure 2:**
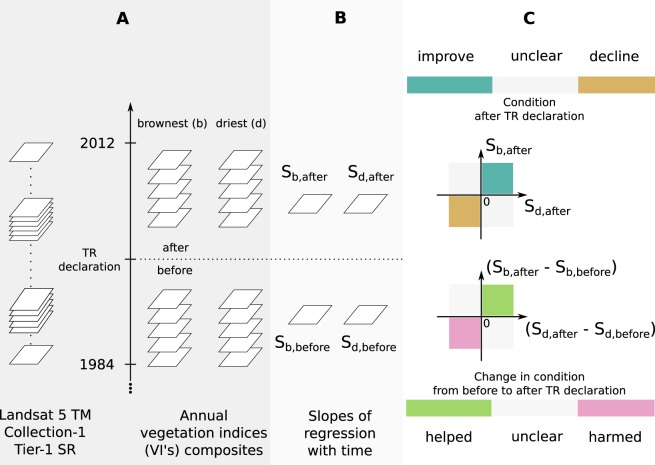
Overview of analysis performed on each PA (TR and WLS) for matched pair comparison of vegetation condition. (**A**) Annual brownest and driest pixel composites are computed from Landsat 5 TM Collection-1 Tier-1 surface reflectance data. (**B**) Epoch-wise slopes of regression with time in time series brownest and driest pixel composites are estimated as ${S}_{b,before}$, ${S}_{b,after}$, ${S}_{d,before}$ and ${S}_{d,after}$, respectively. (**C**) Pixel-wise slopes in the epoch after TR declaration, and their differences between after and before declaration epochs, are compared to (0, 0) to infer categories of vegetation condition and its change.

We computed enhanced vegetation index (EVI)^53^ and normalized difference infrared index (NDII)^54,55^ on cloud- and water-masked Landsat 5 TM Collection-1 Tier-1 surface reflectance dataset^45^. NDII is useful in estimating vegetation canopy moisture content and hence is similar to normalized difference water index (NDWI)^52^. The two differ in the wavelength of the shortwave-infrared band used in their band arithmetic (NDII: 1.55 − 1.75 *μ*m, NDWI: 1.24 *μ*m). We use NDII since Landsat 5 TM lacks a band overlapping 1.24 *μ*m essential for NDWI calculation. We built annual brownest and driest composites from these indices, as yearly minimum EVI and NDII composites, respectively (Fig. 2A). Since these vegetation indices (VIs) are noisy estimates of vegetation condition, their annual minima remain noisy estimates of yearly peak dry season vegetation condition. We mitigated effects of this noise on trend analysis by replacing each year’s annual minima with an average of the least 20% values of the year. On these brownest and driest annual composites, pixel-wise linear trend analysis of their annual change was performed against the pixel-wise date-stamp (fractional years since January 1, 1984) of the annual minimum index values.

Annual brownest and driest composites represent annual peak-summer vegetation conditions, just as the greenest and wettest composites (corresponding to annual maxima in EVI and NDII respectively) represent peak cloud-masked rainy season conditions. An improvement in vegetation condition could reflect in increasing VI trends in either or both these sets of composites, with distinct and unique ecological relevance on the ground. We chose annual composites representative of peak-summer conditions for analysis in this study because, by representing the vegetation at peak temperature and moisture stress within each year, they better capture vegetation condition declines. These, being conservative estimates of vegetation condition and its change, are more relevant from a PA management perspective interested in averting worst-case scenarios and hence in PA effectiveness evaluations. Further, peak summer vegetation conditions (as minima in EVI and NDII and when they occur) can be measured more accurately since summers are mostly cloud-free in India. In contrast, peak wet season condition estimates (as maxima in EVI and NDII and when they occur) are more error-prone because of persistent cloud cover over the sub-continent during the nearly 6 months (June - November) each year when the monsoons bring India most of its rainfall.

#### Statistical analysis

We divided the time period of analysis for each TR and its matched WLS into “before” and “after” declaration epochs based on year of the TR’s declaration. Using the Sen’s slope estimator, a non-parametric repeated medians linear regression method^56,57^, we estimated epoch-wise and pixel-wise slopes in linear rate of change of annual brownest and driest composites with time for all TRs and matched WLSs at 30 m per pixel resolution (Fig. 2B). Any pixel with fewer than 5 valid data points in its time series within an epoch was dropped from the regression in that epoch for having insufficient data. Any PA with more than 20% of its area having insufficient data in an epoch was dropped from its further analysis involving that epoch.

We assessed vegetation condition response to TR declaration by comparing it within matched TR and WLS pairs in two stages. First, in the years after TR declaration (“after” epoch), we compared the slopes of vegetation condition trends in matched TR (impact) and WLS (control) pairs. At each Landsat pixel of PA forest vegetation, we categorized its vegetation condition as “improve”, “decline” or “unclear” based on its regression slopes in brownest and driest composites simultaneously, using the following rules (Fig. 2C):

1 $$\begin{array}{ll}{\rm{improve}}: & ({S}_{b,after} > 0)\,AND\,({S}_{d,after} > 0)\\ {\rm{decline}}: & ({S}_{b,after} < 0)\,AND\,({S}_{d,after} < 0)\\ {\rm{unclear}}: & [({S}_{b,after} > 0)\,AND\,({S}_{d,after} < 0)]\\  & OR\\  & [({S}_{b,after} < 0)\,AND\,({S}_{d,after} > 0)]\end{array}$$

where subscripts ‘*b*’ and ‘*d*’ denote brownest and driest composites, respectively, and ‘*after*’ denotes the after TR declaration epoch. We then arrived at PA-wise summaries of vegetation condition composition by calculating percentage of PA-area under each of these categories. These compositions were compared within matched TR and WLS pairs for insights into TRs in the “after” declaration epoch. A simultaneous positive (increasing) slope in brownness (EVI) and dryness (NDII) measures indicates improving vegetation condition, and simultaneous negative slopes indicates declining condition. The two slopes with opposing signs is an ambiguous scenario, indicating a condition that is unclear.

Second, we assessed vegetation condition response to TR declaration by comparing matched TR and WLS pairs after combining regression slopes from “before” and “after” epochs. At each Landsat pixel of PA forest vegetation, we categorized its vegetation condition change following TR declaration as “helped”, “harmed” or “unclear” based on the *change* in corresponding regression slopes in brownest and driest composites *from* before *to* after declaration epochs. Specifically, we categorized the vegetation condition change from “before” to “after” epochs into “helped”, “harmed” or “unclear” based on the following rules (Fig. 2C):

2 $$\begin{array}{ll}{\rm{helped}}: & ({S}_{b,after}-{S}_{b,before} > 0)\,AND\,({S}_{d,after}-{S}_{d,before} > 0)\\ {\rm{harmed}}: & ({S}_{b,after}-{S}_{b,before} < 0)\,AND\,({S}_{d,after}-{S}_{d,before} < 0)\\ {\rm{unclear}}: & [({S}_{b,after}-{S}_{b,before} > 0)\,AND\,({S}_{d,after}-{S}_{d,before} < 0)]\\  & OR\\  & [({S}_{b,after}-{S}_{b,before} < 0)\,AND\,({S}_{d,after}-{S}_{d,before} > 0)]\end{array}$$

where ‘*b*’ and ‘*d*’ denote brownest and driest composites, respectively, and ‘*after*’ and ‘*before*’ denote the after and before TR declaration epochs respectively. Similar to the analysis for “after” epoch above, we converted these labels into PA-wise summaries of vegetation condition composition by calculating percentage of PA-area under each of these categories. These compositions were then compared within matched TR and WLS pairs for insights into vegetation change in TRs from “before” to “after” epoch. Simultaneous positive differences in corresponding slopes indicates that the vegetation condition was helped by the TR declaration intervention, and simultaneous negative differences suggests that the condition was harmed by the intervention. The two differences with opposing signs is an ambiguous scenario, indicating a condition response that is unclear.

All statistical analyses were carried out on Google Earth Engine^43^. Results were visualized on R Statistical Software^58^ using the dplyr^59^, tidyr^60^, ggplot2^61^ and ggtern^62^ packages and finished in QGIS 2.18 geographic information system application and Inkscape 0.91 image editing software.

## Results

### Tiger Reserve – Wildlife Sanctuary matched pairs

Of the 29 TRs declared before 2000, 25 could be matched with distinct WLSs and were deemed to have sufficient time series data. They spanned 16 states and all 5 management clusters: Central India (CI), Northeastern Hills (NEH), Shivalik Central India (SCI), Shivalik Eastern Ghats (SEG) and Western Ghats (WG) (Fig. 1, Table 1). These 25 matched pairs were analyzed for vegetation condition trends in the “after” epoch. Eight of these pairs (TRs declared between 1992 and 1999), which had sufficient time series data in both “before” and “after” epochs, were analyzed for change in vegetation composition from before to after TR declaration. Of the four TRs dropped from further analysis, two TRs (Buxa and Valmiki) could not be matched with WLSs. Two other TRs (Nagarahole and Bhadra) had insufficient time series data and could not be matched with distinct WLSs.

**Table 1:** The 29 Tiger Reserves in our study with their year of declaration and matched Wildlife Sanctuaries, along with area extents with adequate data availability in their annual driest and brownest composite time series images.

	Id	Tiger Reserve (TR)				Matched Wildlife Sanctuary (WLS)		
		Year decl.	Name	Time series data availability (% area)		Name	Time series data availability (% area)	
				Before	After		Before	After
★	1	1973	Bandipur	0	99.5	Biligiri Ranganatha Temple	0	99.5
★	2	1973	Corbett	0	99.5	Kedarnath	0	81.6
★	3	1973	Kanha	0	99.7	Phen	0	99.7
★	4	1973	Manas	0	99.8	Barail	0	99.7
★	5	1973	Melghat	0	99.7	Yawal	0	99.0
★	6	1973	Palamau	0	99.7	Mahuadanr	0	99.7
★	7	1973	Ranthambore	0	99.8	Ramgarh Vishdhari	0	99.7
★	8	1973	Simlipal	0	99.7	Kuldiha	0	99.7
★	9	1973	Sundarban	0	93.7	Chintamani Kar	0	82.9
★	10	1978	Periyar	0	99.1	Idukki	0	93.4
★	11	1978	Sariska	0	99.8	Jamwa Ramgarh	0	99.8
	12	1982	Buxa			—		
★	13	1982	Indravati	0	99.6	Bhairamgarh	0	99.6
★	14	1982	Nagarjunasagar Srisailam	0	96.7	Gundla Bramheswaram	0	99.6
★	15	1982	Namdapha	0	99.8	Kamlang	0	99.8
★	16	1987	Dudhwa	0	99.8	Sohelwa	0	99.8
★	17	1988	Kalakkad Mundanthurai	0	99.0	Kanyakumari	0	99.3
	18	1989	Valmiki			—		
★	19	1992	Pench MP	0	97.8	Singhori	0	98.2
★ ∧	20	1993	Bandhavgarh	99.7	99.7	Sanjay Dubari	99.6	99.7
★ ∧	21	1993	Tadoba Andhari	99.6	99.7	Umred Karhandla	99.2	99.7
★ ∧	22	1994	Dampa	99.7	99.7	Ngengpui	99.7	99.7
★ ∧	23	1994	Panna	98.7	99.7	Noradehi	99.7	99.7
	24	1998	Bhadra	86.5	8.5	Shettihalli	99.5	0
★ ∧	25	1998	Pench MH	98.3	98.6	Koka	99.7	99.7
★ ∧	26	1999	Nameri	99.8	99.8	Sonai Rupai	99.8	99.8
★ ∧	27	1999	Pakke	99.8	99.8	Sessa	99.8	99.8
★ ∧	28	1999	Satpura	98.2	98.8	Ratapani	99.6	99.6
	29	2000	Nagarahole	99.4	55.9	Shettihalli	99.5	0

Grey cells denote insufficient or unavailable data. ‘★’ denotes matched TR - WLS pairs with sufficient data for vegetation condition analyses after TR declaration (“after” epoch). ‘∧’ denotes the PA pairs with sufficient data for analyses of vegetation condition change from “before” to “after” epochs. Id contains TR identifiers from Fig. 1.

The rainfall, area and separation distances among our study TRs and their candidate WLS pairs, are shown in Supplementary Table S1. Among the 25 matched pairs, the rainfall difference is less than 250 *mm* for 18 pairs, and separating distance is less than 200 *km* for all pairs and less than 100 *km* for 20 pairs.

### Vegetation trends

**Figure 3:**
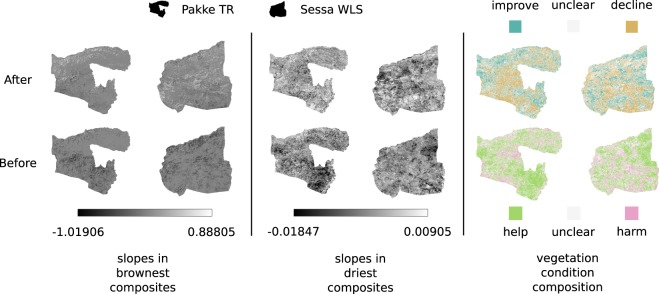
Indicative maps of a matched TR-WLS pair (Pakke TR (declared 1999) and Sessa WLS), showing estimated slopes of temporal change in annual brownest and driest vegetation composites in “before” and “after” epochs, and the inferred vegetation conditions.

For each PA in the 25 matched TR - WLS pairs, we estimated the slope of linear trends in the annual brownest and driest pixel composites time series using the Sen’s slope estimator in “before” and “after” epochs separately (Fig. 3). By calculating and comparing areas of positive and negative slopes in the “after” epoch, as well as increasing and decreasing slopes from “before” to “after” epochs, we assessed the effect of protection elevation of TRs on their vegetation condition (Fig. 2C).

#### TRs after declaration

**Figure 4:**
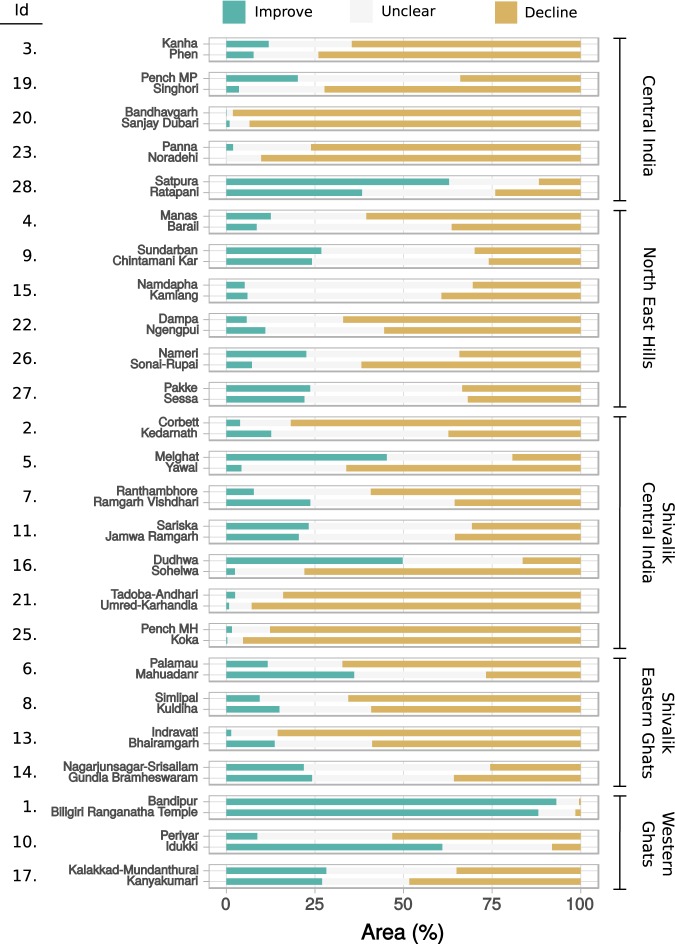
Vegetation condition compositions of TRs (top in each pair) and their matched WLS (bottom in each pair), in the years after the TR’s declaration. The PAs are grouped by landscape management clusters (denoted on the right) and arranged within each cluster in chronological order of TR declaration year. The Id’s (left) are matched TR-WLS pair identifiers in Table 1 and on the study area map in Fig. 1.

In the years after TR declaration, vegetation condition improved over more than 50% of their areas in 2 out of 25 TRs: Satpura TR (CI) and Bandipur TR (WG), with Bandipur TR showing improvement over 90% of its area (Fig. 4). In the same epoch, vegetation condition declined over more than 50% of their areas in 13 (CI: 3, NEH: 2, SCI: 4, SEG: 3 and WG: 1) out of 25 TRs, with declines over more than 75% of their areas in 6 of them: Bandhavgarh TR (CI), Panna TR (CI), Corbett TR (SCI), Pench MH TR (SCI), Tadoba Andhari TR (SCI) and Indravati TR (SEG).

When compositions were compared within matched TR - WLS pairs, 12 (CI: 4, NEH: 1, SCI: 5 and WG: 2) out of 25 TRs showed, simultaneously, greater percentage of area in improvement and lesser in decline compared to their matched WLSs, indicating better overall vegetation condition trends in these TRs. In contrast, worse vegetation condition was indicated in 8 (CI: 1, NEH: 1, SCI: 2, SEG: 3 and WG: 1) out of 25 TRs where they showed lower percentage of areas in improvement and greater percentage in decline as compared to their matched WLSs. This comparison was ambiguous in the remaining 5 TRs (NEH: 4, SEG: 1) where their areas under both improvement and decline were simultaneously greater or lesser than in their matched WLSs.

#### Extent of change in vegetation condition in TRs vs. WLSs

To understand the strength of effects of protection level elevation on vegetation condition responses in the “after” epoch, we investigated the magnitudes of differences in vegetation condition compositions between TRs and their matched WLSs. Eleven of 25 TRs in our study were substantially different from their matched WLS pairs (i.e., at least 15% difference in percentage of areas under improvement or decline). Of these, 5 TRs (CI: 2, NEH: 1, SCI: 2) showed better vegetation conditions and 5 TRs (SCI: 2, SEG: 2, WG: 1) showed worse vegetation conditions than their matched WLSs. The comparison was ambiguous in 1 TR (Sundarban TR (NEH)), whose area under both improvement and decline were simultaneously greater than in its matched WLS (Chintamani Kar WLS). The remaining 14 TRs in our study – over 50% of them – showed little difference from their matched WLSs (i.e., less than 15% difference in percentage of areas under improvement and decline). Plots of PA-wise vegetation condition compositions in ternary space (Supplementary Material Fig. S1) depict these extents of difference: the farther apart the TRs and WLSs are in ternary space the greater the extent of difference in their vegetation conditions.

#### Change in TRs from before to after declaration

**Figure 5:**
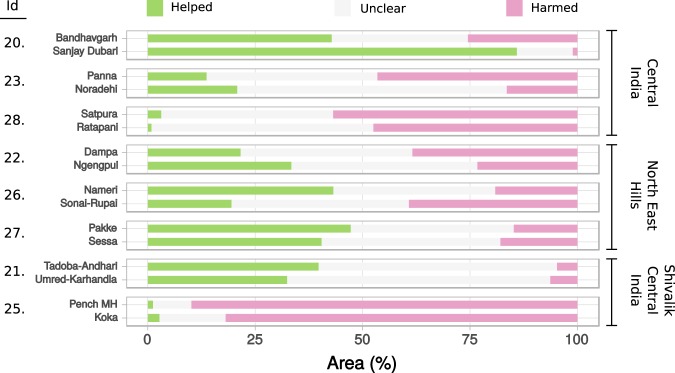
Composition of vegetation condition change from before to after TR declaration, in TRs (top, in each pair) and their matched WLSs (bottom, in each pair). The matched pairs are grouped by the TR’s landscape management cluster and arranged within each cluster in chronological order of TR declaration. The Id’s (left) are matched TR-WLS pair identifiers in Table 1 and on the study area map in Fig. 1.

For the eight matched TR - WLS pairs with sufficient time series data in both “before” and “after” epochs, we computed percentage areas of vegetation condition being helped or harmed based on the differences in slopes of vegetation condition change from their “before” to “after” epochs. Vegetation in more than 25% of the area was helped in 4 (CI: 1, NEH: 2, SCI: 1) out of 8 TRs. Five (CI: 3, NEH: 1, SCI: 1) out of 8 TRs saw vegetation harmed in more than 25% of their areas, with 2 of these (Satpura TR (CI), Pench MH TR (SCI)) seeing vegetation in more than 50% of their area harmed (Fig. 5).

When these compositions of vegetation change from “before” to “after” epochs were compared within matched TR - WLS pairs, 3 (NEH: 2, SCI: 1) out of 8 TRs showed greater percentage areas helped and lesser harmed compared to their matched WLSs suggesting a positive effect of TRs’ greater protection status on their vegetation condition. However, 4 (CI: 2, NEH: 1, SCI: 1) out of 8 TRs showed lower percentage areas helped and greater harmed when compared to their matched WLSs, suggesting a negative effect of TRs’ higher protection on their overall vegetation condition. This comparison was ambiguous with one of the TRs (Satpura (CI)) due to its percentage areas of being helped and harmed being more than those in its matched WLS (Ratapani WLS).

#### Extent of change in vegetation condition in TRs vs. WLSs

Along similar lines as the assessment of extent of differences in vegetation trends compositions in the “after” epoch, here too we investigated the magnitude of differences within matched TR - WLS pairs in percentage area compositions of being helped and harmed. These compositions were substantially different (i.e., at least 15% difference in percentage of areas helped or harmed) within 4 (CI: 2, NEH: 2) of 8 TRs compared to their matched WLSs. Of these, one TR (Nameri TR (NEH)) showed a greater percentage area helped and lesser harmed compared to its matched WLS, suggesting a substantial positive effect of TR’s greater protection status on its vegetation condition. In contrast, 3 TRs (CI: 2, NEH: 1) showed a lesser percentage area helped and greater harmed, suggesting a substantial negative effect. The remaining 4 TRs (CI: 1, NEH: 1, SCI: 2) showed little difference (i.e., less than 15% difference in percentage of areas helped and harmed) from their matched WLSs. Ternary plots of these PA-wise vegetation condition composition changes (Supplementary Material Fig. S2) capture these extents of difference: the farther apart the TRs and WLSs are in ternary space the greater the extent of difference in their vegetation conditions.

## Discussion

In this study, we used annual brownest and driest pixel composites from Landsat 5 TM imagery of 25 Indian TRs and their matched WLSs to assess their vegetation condition changes over nearly three decades from 1984 to 2012. This framework allowed us to evaluate effectiveness of protection elevation of TRs in improving their forest vegetation condition. We found that only a minority (<50%) of TRs had vegetation over majority extents showing improvement after TR declaration. About half of the TRs showed moderate extents of vegetation being helped following TR declaration, while a similar proportion of TRs showed majority extents harmed. Upon investigating how these broad trends compared across two protection levels (which also correlates with use of umbrella species conservation approach), we found that roughly half of the TRs (higher protection) were worse than or similar to their matched WLSs (lower protection), in their epoch after declaration. Likewise, roughly half of the TRs investigated for change from before to after declaration were found to be worse than or similar to their matched WLSs. These results suggest that protection elevation alone may be insufficient to improve their forests’ vegetation condition, thus challenging the effectiveness of greater protection and management resources alone in safeguarding long-term viability of habitats of the tiger as a threatened species. Explaining these patterns and understanding their drivers needs further investigation since beneficial effects of designating and managing TRs may be swamped by other stressors operating at local, regional, landscape and continental scales, thus leaving their vegetation condition responses similar to those in their less-protected neighbouring similar PAs without the tiger-centric conservation management. These stressors include PA-specific habitat degradation and human developmental pressures in their vicinity as well as abiotic factors such as changing temperature and rainfall patterns^63–65^.

By bringing a vegetation condition perspective to forest assessment in PAs, this study adds to the understanding of the state of forests in India’s TRs where forest extent is thought to have remained largely stable in recent years^40^. Additionally, a before-after control-impact style comparison framework involving long time series data allowed for inferring effectiveness of protection elevation on vegetation condition preservation. However, limitations to our inferences exist due to difficulties in ascertaining ground-level vegetation condition – and hence habitat quality – from remotely sensed VIs alone, in addition to known issues in Landsat imagery^66^. First, VIs as measures of on-ground vegetation quality are susceptible to noise and hence render vegetation change detection solely from remotely sensed data challenging^67,68^. We help mitigate this in our study by calculating annual brownest and driest composites as averages of a year’s least 20% of the index values instead of as absolute minima, and further gains may be had from extensive calibration of indices and validation with ground data. Extending our work to explicitly include burnt area^69^ and forest loss^70^ maps will help separate vegetation condition changes in regenerating forest tracts from those in persistent forests, which have distinct ecological implications on ground. Second, elevation gradients and complex terrains add uncertainty to VIs-based vegetation condition assessments^44^. Since this tends to be severe at pronounced ridges and valleys in mountainous regions^71,72^ and those features form relatively small portions of most PAs in our study, we have not explicitly addressed this source of uncertainty in our results. Third, loss of vegetation cover to construction of roads and other man-made features could induce errors in time series trend analysis of VIs. Since land cover conversion away from forest within all PAs in India is strictly regulated by law, they are restricted to a tiny portion of their total area even where they are allowed and hence are unlikely to greatly alter PA-level percentage-area summaries such as the ones in this study. Fourth, ecologically relevant habitat condition is subjective to focal taxon even within the umbrella species framework, and is a complex function of multiple biotic and abiotic factors in forests at multiple spatial and temporal scales. Remotely sensed VIs can be hard pressed to capture these complexities^73–75^. Fifth, understanding the important drivers behind the changes reported by this study is critical for informing changes in on-ground management practices, but that is beyond the scope of this study. Additionally, more refined, stringent and management-oriented criteria for matching PAs – by incorporating eg. PA size, surrounding land-use and recent changes in climatic conditions – can help make such results more relevant from a TR management perspective. This, as well as the rest of the limitations above, can be mitigated by further and focused on-ground investigations and monitoring for vegetation condition and composition. Our PA-wise maps of remote sensed forest vegetation condition trend summaries of nearly 3 decades at 30 m/pixel resolution can augment such habitat monitoring efforts as well as enhance planning and prioritization of on-ground research and management action.

Calls for quantitative evaluation of effectiveness of biodiversity conservation measures in general and of PAs in particular have been gaining strength among the conservation community in recent years^13,76^ and the value addition by satellite remote sensed data in such endeavours is well acknowledged^77,78^. The growth of satellite data and derived products in the public domain, together with the advent of the free-to-use Google Earth Engine cloud computing platform^43^, has catalyzed earth observation research. In this study, we demonstrated an application of before-after control-impact comparison^41^ using these open data and computation resources in evaluating effectiveness of protection elevation of PAs in conserving forest vegetation condition in India’s TRs. Such an approach could effectively augment ongoing national-level periodic assessments of natural resource management and conservation measures. It could also help in continuous monitoring and adaptive management at large spatial and management scales by assisting in planning ground data collection for essential biodiversity variables^79^, which could in turn alleviate the current difficulties in evaluation due to a paucity of relevant data.

## Supplementary information

Supplementary material

## Data Availability

Our data and associated computer code and results from this study are available at 10.17632/6jhr4xfs3x.1. A web application https://pradeepkoulgi.users.earthengine.app/view/india-tr-condition visualizes these results. WorldClim Climatology V1^46^ and Landsat 5 TM Collection 1 Tier 1 surface reflectance^45^ datasets are open datasets and are available on the Google Earth Engine public data catalog (https://developers.google.com/earth-engine/datasets/catalog/WORLDCLIM_V1_MONTHLY and https://developers.google.com/earth-engine/datasets/catalog/LANDSAT_LT05_C01_T1_SR).
