# Supplementary material for: Extensive vegetation browning and drying in forests of India’s Tiger Reserves
Source: Sci Rep. 2019 Oct 18;9:14976. doi: 10.1038/s41598-019-51118-8 (PMC6802094; doi:10.1038/s41598-019-51118-8)
Supplement: Supplementary file 1 — Supplementary material [file 41598_2019_51118_MOESM1_ESM.pdf]

Supplementary material for: Extensive vegetation browning and drying in forests of India's Tiger Reserves

Pradeep S. Koulgi*[1], Nicholas Clinton †[2], and Krithi K. Karanth‡[1,3]

[1]Centre for Wildlife Studies, Bengaluru, India
[2]Google, Inc., Mountain View, CA 94043, USA
[3]Duke University, Durham, North Carolina, USA

This supplementary material contains the following 3 items:

- **Figure S1.** After Tiger Reserve (TR) declaration: Vegetation condition compositions comparison in ternary compositional space among matched TR - Wildlife Sanctuary (WLS) pairs.
- **Figure S2.** Change from before to after TR declaration: Vegetation condition compositions comparison in ternary compositional space among matched TR - WLS pairs.
- **Table S1.** The 29 Tiger Reserves (TRs) in our study, their candidate and selected matched Wildlife Sanctuaries (WLSs), along with TR declaration years, state located in and average annual rainfall and separating distance attributes for matching.

*Corresponding author. Email: pradeep.koulgi@gmail.com
†nclinton@google.com
‡krithi.karanth@cwsindia.org

Figure S1. After Tiger Reserve (TR) declaration: Vegetation condition compositions comparison in ternary compositional space among matched TR - Wildlife Sanctuary (WLS) pairs.

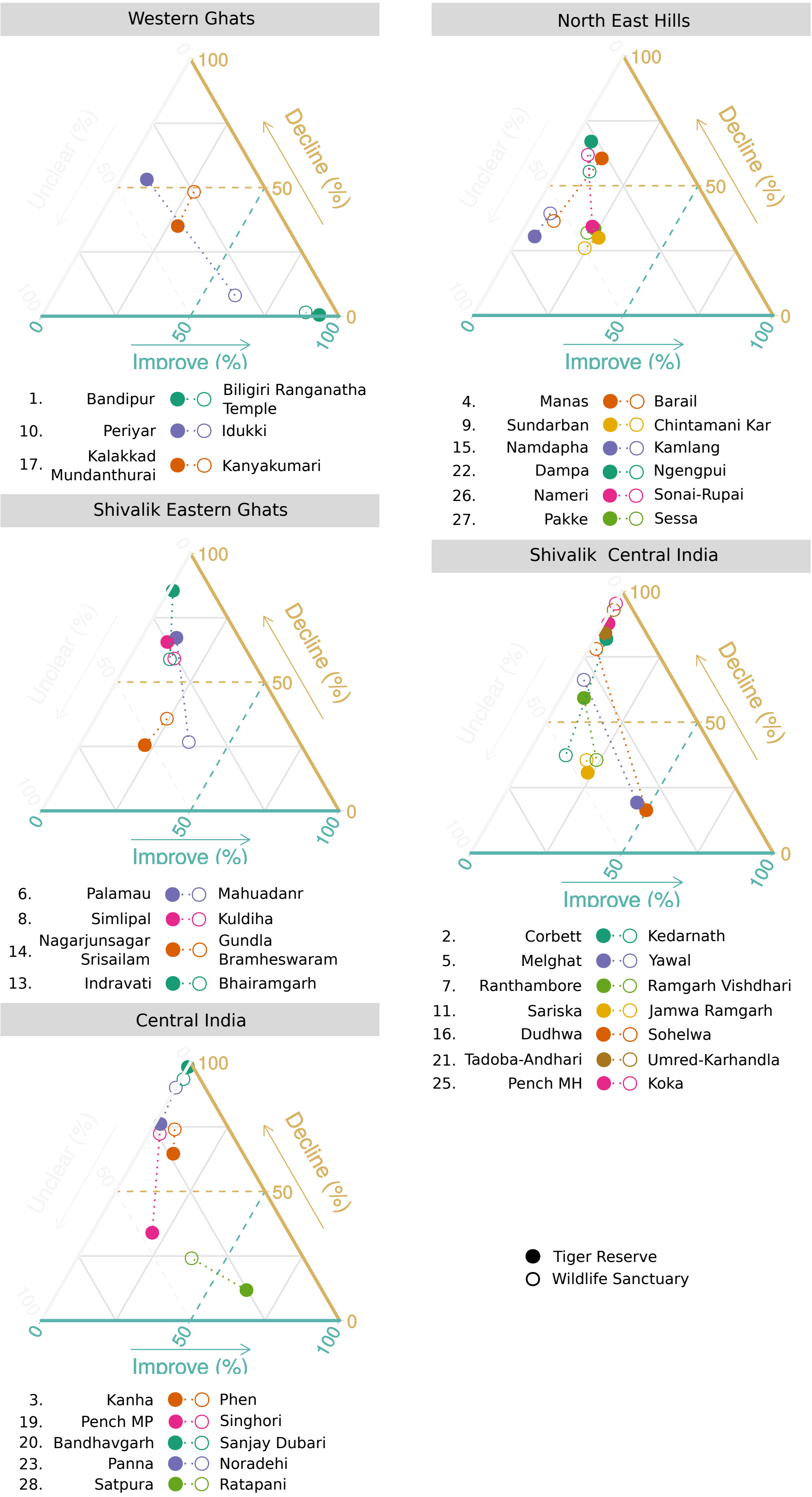

Figure S1: Compositions of vegetation condition trends in TRs and WLSs in the epoch after TR declaration shown in ternary plots arranged by landscape management clusters. Filled circles are TRs and open circles are WLSs. Dotted lines connect TRs with their matched WLS pairs.

Figure S2. Change from before to after TR declaration: Vegetation condition compositions comparison in ternary compositional space among matched TR - WLS pairs.

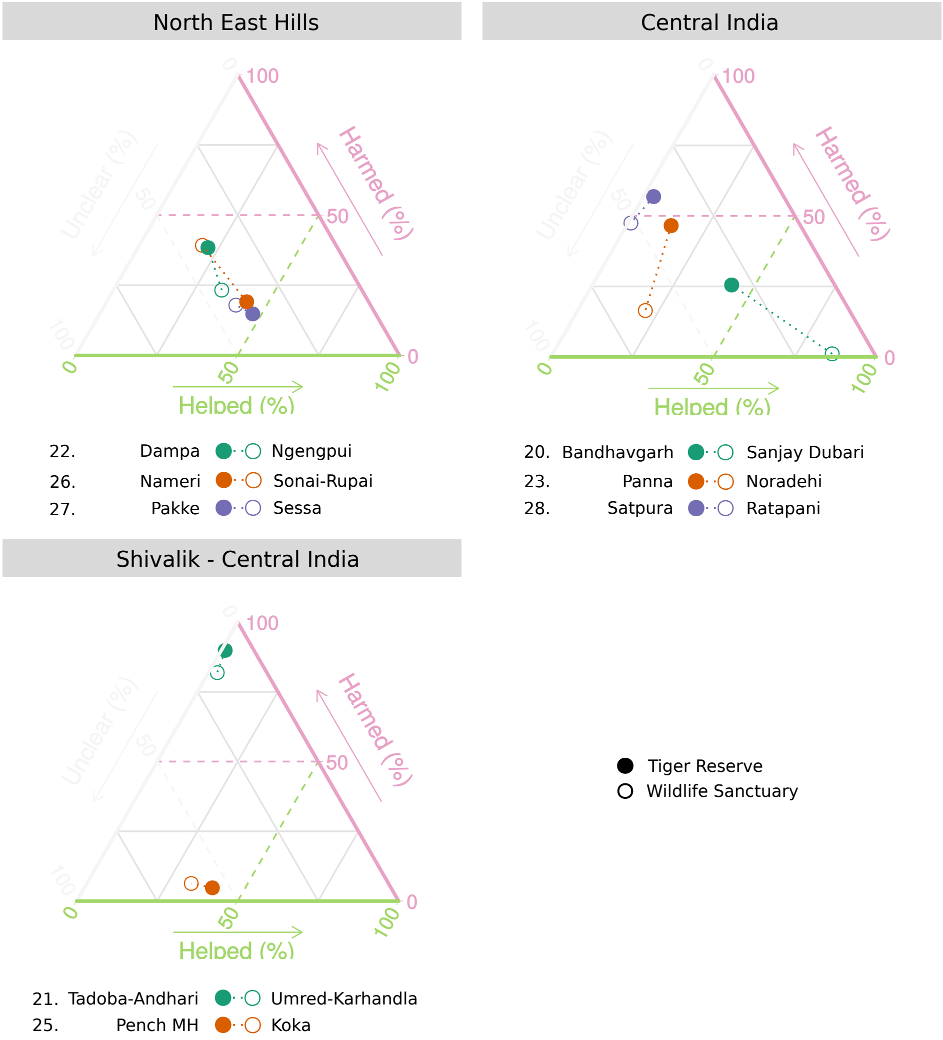

Figure S2: Compositions of vegetation condition change from before to after TR declaration, in matched TR - WLS pairs shown in ternary plots arranged by landscape management clusters. Filled circles are TRs, open circles are WLSs. Dotted lines connect TRs with their matched WLS pairs.

Table S1. The 29 Tiger Reserves (TRs) in our study, their candidate and selected matched Wildlife Sanctuaries (WLSs), along with TR declaration years, state located in and average annual rainfall and separating distance attributes for matching.

Id	Tiger Reserve (TR)					List of candidate Wildlife Sanctuaries for matching (sorted by distance from TR)				Matched Wildlife Sanctuary		
	**Name**	**State**	**Year**	**Area** (km^2)	**Rainfall** (mm/yr)	**Name**	**Distance** (km)	**Rainfall diff.** (mm/yr)	**Area** (km^2)	**Name**	**Distance** (km)	**Rainfall diff.** (mm/yr)
1	Bandipur	Karnataka	1973	931.37	1217.18	[Biligiri Ranganatha Temple, Shettihalli, Chinchioli]	[17.80, 216.35, 612.16]	[476.70, 325.77, 278.40]	[649.11, 575.59, 159.41]	Biligiri Ranganatha Temple	17.81	476.71
2	Corbett	Uttarakhand	1973	815.45	1607.52	[Kedarnath, Askot Musk Deer]	[95.72, 114.96]	[303.64, 116.78]	[1054.65, 477.68]	Kedarnath	95.72	303.65
3	Kanha	Madhya Pradesh	1973	930.24	1376.76	[Phen, Noradehi, Sanjay Dubari, Singhori, Ratapani, Bagdara, Kheoni, Karera, Ghatigaon, Sardarpur]	[6.57, 144.07, 187.81, 243.40, 259.44, 287.57, 362.82, 417.69, 478.00, 570.28]	[89.99, 187.98, 279.46, 129.28, 121.45, 420.07, 329.60, 476.49, 486.27, 484.19]	[109.61, 1306.85, 808.02, 305.50, 818.17, 308.49, 78.71, 200.81, 359.76, 175.13]	Phen	6.58	90.00
4	Manas	Assam	1973	2913.59	3491.52	[Barail]	[196.17]	[255.77]	[353.26]	Barail	196.18	255.78

Id	Tiger Reserve (TR)					List of candidate Wildlife Sanctuaries for matching (sorted by distance from TR)				Matched Wildlife Sanctuary		
	**Name**	**State**	**Year**	**Area** (km^2)	**Rainfall** (mm/yr)	**Name**	**Distance** (km)	**Rainfall diff.** (mm/yr)	**Area** (km^2)	**Name**	**Distance** (km)	**Rainfall diff.** (mm/yr)
5	Melghat	Maharashtra	1973	1589.28	1309.55	[Yawal, Bor, Painganga, Umred-Karhandla, Koka, New Nagzira, Nagzira, Nawegaon, Chaprala, Tungareshwar, Kalsubai Harishchandragad, Bhamragarh]	[73.83, 141.00, 195.96, 219.24, 243.85, 254.30, 266.37, 293.96, 320.82, 340.52, 346.34, 400.34]	[437.84, 268.04, 242.00, 1.36, 1.32, 51.32, 67.96, 215.70, 165.30, 268.51, 418.41, 293.28]	[224.72, 82.60, 377.64, 189.53, 94.71, 135.00, 158.75, 114.85, 135.77, 144.21, 281.88, 108.61]	Yawal	73.83	437.84
6	Palamau	Jharkhand	1973	1120.39	1217.22	[Mahuadanr, Lawalong, Palkot, Gautam Budha, Hazaribagh, Koderma, Dalma]	[0.0, 29.96, 57.17, 87.10, 87.60, 137.73, 186.31]	[56.33, 79.34, 274.13, 204.76, 68.50, 213.41, 91.84]	[514.81, 492.69, 753.23, 141.90, 225.10, 158.48, 237.43]	Mahuadanr	0.00	56.34

Id	Tiger Reserve (TR)					List of candidate Wildlife Sanctuaries for matching (sorted by distance from TR)				Matched Wildlife Sanctuary		
	**Name**	**State**	**Year**	**Area** (km^2)	**Rainfall** (mm/yr)	**Name**	**Distance** (km)	**Rainfall diff.** (mm/yr)	**Area** (km^2)	**Name**	**Distance** (km)	**Rainfall diff.** (mm/yr)
7	Ranthambhore	Rajasthan	1973	1294.33	789.33	[Ramgarh Vishdhari, Bandh Baratha, Ramsagar, Jawahar Sagar, Jamwa Ramgarh, Darrah, Shergarh, Bhensrodgarh, Bassi, Todgarh Raoli, Kumbhalgarh, Sitamata, Jaisamand, Phulwari Ki Nal, Mount Abu]	[41.82, 42.41, 52.07, 96.98, 97.20, 107.84, 115.99, 122.88, 160.31, 215.73, 216.74, 242.95, 293.33, 313.98, 369.00]	[51.12, 65.47, 45.06, 43.97, 183.31, 52.40, 85.30, 68.44, 45.344, 321.62, 272.94, 17.72, 193.25, 75.36, 267.22]	[295.44, 189.71, 150.51, 204.21, 309.86, 327.70, 103.77, 208.04, 152.27, 437.32, 973.66, 420.58, 152.01, 1825.19, 365.99]	Ramgarh Vishdhari	41.83	51.12

Id	Tiger Reserve (TR)					List of candidate Wildlife Sanctuaries for matching (sorted by distance from TR)				Matched Wildlife Sanctuary		
	**Name**	**State**	**Year**	**Area** (km^2)	**Rainfall** (mm/yr)	**Name**	**Distance** (km)	**Rainfall diff.** (mm/yr)	**Area** (km^2)	**Name**	**Distance** (km)	**Rainfall diff.** (mm/yr)
8	Simlipal	Orissa	1973	2361.14	1476.53	[Kuldiha, Bhitarkanika, Gahirmatha (Marine), Hadgarh, Chandaka Dampara, Satkosia Gorge, Khalasuni, Badarma, Baisipalli, Balukhand Konark, Debrigarh, Kothagarh, Lakhari Valley, Karlapat, Sunabeda]	[16.89, 90.88, 108.61, 121.96, 125.05, 152.20, 162.41, 169.01, 172.02, 174.40, 239.77, 290.59, 297.48, 369.98, 382.89]	[92.39, 19.87, 0.30, 0.23, 8.21, 106.83, 40.17, 81.36, 97.33, 107.40, 36.59, 157.08, 246.62, 189.83, 86.42]	[228.99, 734.33, 1365.70, 197.28, 193.99, 659.93, 212.47, 461.19, 403.58, 85.84, 333.72, 463.51, 152.26, 150.39, 634.73]	Kuldiha	16.90	92.40
9	Sundarban	West Bengal	1973	2623.64	1745.95	[Chintamani Kar]	[0.0]	[94.35]	[1565.03]	Chintamani Kar	0.00	94.35
10	Periyar	Kerala	1978	796.08	1967.71	[Idukki, Shendurney, Pepara, Chinnar, Neyyar, Parambikulam, Wayanad]	[16.95, 37.63, 65.41, 70.62, 71.16, 87.23, 235.79]	[307.53, 314.07, 422.89, 454.02, 492.85, 147.40, 219.53]	[181.23, 162.76, 79.57, 79.99, 110.01, 299.13, 363.16]	Idukki	16.96	307.53

Id	Tiger Reserve (TR)					List of candidate Wildlife Sanctuaries for matching (sorted by distance from TR)				Matched Wildlife Sanctuary		
	**Name**	**State**	**Year**	**Area** (km^2)	**Rainfall** (mm/yr)	**Name**	**Distance** (km)	**Rainfall diff.** (mm/yr)	**Area** (km^2)	**Name**	**Distance** (km)	**Rainfall diff.** (mm/yr)
11	Sariska	Rajasthan	1978	878.62	708.30	[Jamwa Ramgarh, Bandh Baratha, Ramsagar, Ramgarh Vishdhari, Jawahar Sagar, Kumbhalgarh, Darrah, Todgarh Raoli, Bhensrodgarh, Bassi, Shergarh, Sitamata, Phulwari Ki Nal, Jaisamand, Mount Abu]	[0.0, 83.21, 117.79, 162.97, 222.65, 232.74, 232.75, 238.87, 243.10, 255.15, 255.62, 347.26, 378.29, 385.45, 428.25]	[102.28, 15.55, 35.95, 29.90, 125.00, 191.91, 133.43, 240.59, 149.47, 35.68, 166.33, 98.75, 156.39, 112.22, 348.24]	[309.86, 189.71, 150.51, 95.44, 204.21, 973.66, 327.70, 437.32, 208.04, 152.27, 103.77, 420.58, 1825.19, 152.01, 365.99]	Jamwa Ramgarh	0.00	102.29
12	Buxa	West Bengal	1982			—				—		

Id	Tiger Reserve (TR)					List of candidate Wildlife Sanctuaries for matching (sorted by distance from TR)				Matched Wildlife Sanctuary		
	**Name**	**State**	**Year**	**Area** (km^2)	**Rainfall** (mm/yr)	**Name**	**Distance** (km)	**Rainfall diff.** (mm/yr)	**Area** (km^2)	**Name**	**Distance** (km)	**Rainfall diff.** (mm/yr)
13	Indravati	Chattisgarh	1982	1354.57	1615.72	[Bhairamgarh, Pamed Wild Buffalo, Sitanadi, Udanti Wild Buffalo, Barnawapara, Bhoramdev, Sarangarh-Gomardha, Achanakmar, Tamor Pingla, Badalkhol, Semarsot]	[22.47, 57.31, 149.15, 176.52, 275.62, 288.53, 340.36, 347.54, 502.23, 506.61, 535.59]	[88.35, 219.72, 233.93, 248.87, 321.79, 418.20, 275.02, 344.02, 403.47, 165.52, 354.99]	[142.09, 834.55, 574.98, 293.85, 343.16, 177.51, 290.69, 554.61, 950.93, 112.62, 593.90]	Bhairamgarh	22.48	88.36

**Id**	**Tiger Reserve (TR)**					**List of candidate Wildlife Sanctuaries for matching (sorted by distance from TR)**				**Matched Wildlife Sanctuary**		
	**Name**	**State**	**Year**	**Area** (km^2)	**Rainfall** (mm/yr)	**Name**	**Distance** (km)	**Rainfall diff.** (mm/yr)	**Area** (km^2)	**Name**	**Distance** (km)	**Rainfall diff.** (mm/yr)
14	Nagarjunsagar-Srisailam	Andhra Pradesh	1982	5200.74	711.13	[Gundla Bramheswaram, Sri Lankamalleswaram, Pakhal, Sri Penusila Narasimha, Krishna, Kinnersani, Kolleru, Pocharam, Eturnagaram, Kawal, Kaundinya, Coringa]	[0.0, 128.82, 135.23, 135.75, 156.49, 165.87, 169.83, 182.98, 190.15, 264.84, 281.63, 296.27]	[42.19, 36.10, 343.39, 98.76, 311.99, 396.49, 249.38, 342.81, 187.06, 357.33, 17.88, 365.47]	[2319.30, 486.39, 1307.47, 1274.62, 318.95, 649.91, 178.16, 144.57, 950.15, 977.47, 831.38, 250.63]	Gundla Bramheswaram	0.00	42.19
15	Namdapha	Arunachal Pradesh	1982	1876.78	2519.95	[Kamlang, Mehao, Itanagar, Sessa]	[0.31, 61.18, 255.54, 349.93]	[446.11, 16.75, 204.21, 200.89]	[703.91, 297.86, 164.27, 253.36]	Kamlang	0.31	446.11
16	Dudhwa	Uttar Pradesh	1987	1558.54	1470.51	[Sohelwa, Hastinapur, Sohagibarwa]	[55.05, 208.75, 215.04]	[9.11, 483.71, 13.43]	[422.88, 1912.32, 514.15]	Sohelwa	55.06	9.11

Id	Tiger Reserve (TR)					List of candidate Wildlife Sanctuaries for matching (sorted by distance from TR)				Matched Wildlife Sanctuary		
	**Name**	**State**	**Year**	**Area** (km^2)	**Rainfall** (mm/yr)	**Name**	**Distance** (km)	**Rainfall diff.** (mm/yr)	**Area** (km^2)	**Name**	**Distance** (km)	**Rainfall diff.** (mm/yr)
17	Kalakkad-Mundanthurai	Tamil Nadu	1988	911.03	1435.31	[Kanyakumari, Srivilliputhur, Megamalai, Kodailkanal, Indira Gandhi (Annamalai)]	[0.0, 57.53, 76.41, 136.35, 150.73]	[207.39, 246.44, 191.00, 27.71, 193.22]	[465.38, 456.73, 163.69, 449.51, 897.21]	Kanyakumari	0.00	207.39
18	Valmiki	Bihar	1989			—				—		
19	Pench MP	Madhya Pradesh	1992	438.85	1085.28	[Noradehi, Phen, Singhori, Ratapani, Kheoni, Sanjay Dubari, Bagdara, Karera, Sardarpur, Kuno-Palpur, Gandhi Sagar, Ghatigaon]	[135.12, 156.82, 172.10, 173.55, 246.29, 304.63, 412.57, 418.43, 441.93, 465.66, 465.79, 484.20]	[103.50, 201.48, 162.19, 170.03, 38.12, 12.02, 128.59, 185.00, 192.71, 306.01, 229.40, 194.79]	[1306.85, 109.61, 305.50, 818.17, 78.71, 808.02, 308.49, 200.81, 175.13, 326.49, 434.43, 359.76]	Singhori	172.10	162.20

Id	Tiger Reserve (TR)					List of candidate Wildlife Sanctuaries for matching (sorted by distance from TR)				Matched Wildlife Sanctuary		
	**Name**	**State**	**Year**	**Area** (km^2)	**Rainfall** (mm/yr)	**Name**	**Distance** (km)	**Rainfall diff.** (mm/yr)	**Area** (km^2)	**Name**	**Distance** (km)	**Rainfall diff.** (mm/yr)
20	Bandhavgarh	Madhya Pradesh	1993	731.42	1174.21	[Sanjay Dubari, Phen, Bagdara, Noradehi, Singhori, Ratapani, Karera, Ghatigaon, Kheoni, Kuno-Palpur, Gandhi Sagar, Sardarpur]	[33.87, 120.08, 137.21, 142.43, 260.83, 286.61, 334.61, 378.58, 397.62, 415.00, 515.26, 602.85]	[76.90, 112.55, 217.52, 14.57, 73.26, 81.10, 273.93, 283.72, 127.05, 394.94, 318.33, 281.64]	[808.02, 109.61, 308.49, 1306.85, 305.50, 818.17, 200.81, 359.76, 78.71, 326.49, 434.43, 175.13]	Sanjay Dubari	33.87	76.91
21	Tadoba-Andhari	Maharashtra	1993	615.21	1362.96	[Umred-Karhandla, Chaprala, Bor, Koka, Nawegaon, New Nagzira, Nagzira, Bhamragarh, Painganga, Yawal, Tungareshwar, Kalsubai Harishchandragad]	[42.42, 52.49, 79.52, 88.52, 97.53, 101.23, 111.36, 128.97, 132.95, 356.15, 558.88, 566.31]	[54.77, 111.88, 321.45, 52.08, 162.28, 2.09, 14.55, 239.86, 295.42, 491.25, 321.92, 365.00]	[189.53, 135.77, 82.60, 94.71, 114.85, 135.00, 158.75, 108.61, 377.64, 224.72, 144.21, 281.88]	Umred-Karhandla	42.43	54.78

Id	Tiger Reserve (TR)					List of candidate Wildlife Sanctuaries for matching (sorted by distance from TR)				Matched Wildlife Sanctuary		
	**Name**	**State**	**Year**	**Area** (km^2)	**Rainfall** (mm/yr)	**Name**	**Distance** (km)	**Rainfall diff.** (mm/yr)	**Area** (km^2)	**Name**	**Distance** (km)	**Rainfall diff.** (mm/yr)
22	Dampa	Mizoram	1994	293.64	2460.41	[Ngengpui]	[107.52]	[188.49]	[80.37]	Ngengpui	107.53	188.49
23	Panna	Madhya Pradesh	1994	611.24	1081.89	[Noradehi, Sanjay Dubari, Karera, Singhori, Bagdara, Ghatigaon, Ratapani, Phen, Kuno-Palpur, Kheoni, Gandhi Sagar, Sardarpur]	[102.01, 151.69, 187.26, 210.58, 219.32, 232.38, 241.05, 252.90, 269.72, 342.27, 395.52, 527.93]	[106.89, 15.41, 181.61, 165.58, 125.20, 191.40, 173.42, 204.87, 302.61, 34.73, 226.01, 189.32]	[1306.85, 808.02, 200.81, 305.50, 308.49, 359.76, 818.17, 109.61, 326.49, 78.71, 434.43, 175.13]	Noradehi	102.02	106.89
24	Bhadra	Karnataka	1998	541.93	1502.42	[Shettihalli]	[5.21]	[40.52]	[575.59]	Shettihalli	5.22	40.53

Id	Tiger Reserve (TR)					List of candidate Wildlife Sanctuaries for matching (sorted by distance from TR)				Matched Wildlife Sanctuary		
	**Name**	**State**	**Year**	**Area** (km^2)	**Rainfall** (mm/yr)	**Name**	**Distance** (km)	**Rainfall diff.** (mm/yr)	**Area** (km^2)	**Name**	**Distance** (km)	**Rainfall diff.** (mm/yr)
25	Pench MH	Maharashtra	1998	534.22	1045.90	[Koka, New Nagzira, Bor, Umred-Karhandla, Nagzira, Nawegaon, Chaprala, Painganga, Dhyanganga, Yawal, Aner Dam, Gautala-Autramghat, Jaikwadi, Tungareshwar]	[52.02, 60.81, 63.77, 67.40, 68.12, 102.08, 206.33, 226.87, 289.85, 322.90, 402.34, 413.24, 441.14, 579.48]	[264.97, 314.97, 4.39, 262.28, 331.61, 479.35, 428.95, 21.64, 297.54, 174.18, 324.12, 257.99, 377.02, 4.85]	[94.71, 135.00, 82.60, 189.53, 158.75, 114.85, 135.77, 377.64, 217.65, 224.72, 93.11, 293.41, 240.91, 144.21]	Koka	52.03	264.98
26	Nameri	Assam	1999	385.83	2310.54	[Sonai-Rupai, Amchang, Dihing Patkai]	[7.78, 110.40, 235.40]	[114.79, 380.11, 175.79]	[225.71, 79.60, 103.05]	Sonai-Rupai	7.78	114.80
27	Pakke	Arunachal Pradesh	1999	648.64	2258.60	[Sessa, Itanagar, Tale Valley, Mehao, Kamlang]	[0.0, 38.94, 88.07, 287.98, 331.21]	[60.46, 57.14, 340.56, 278.10, 184.75]	[253.36, 164.27, 347.18, 297.86, 703.91]	Sessa	0.00	60.46

Id	Tiger Reserve (TR)					List of candidate Wildlife Sanctuaries for matching (sorted by distance from TR)				Matched Wildlife Sanctuary		
	**Name**	**State**	**Year**	**Area** (km^2)	**Rainfall** (mm/yr)	**Name**	**Distance** (km)	**Rainfall diff.** (mm/yr)	**Area** (km^2)	**Name**	**Distance** (km)	**Rainfall diff.** (mm/yr)
28	Satpura	Madhya Pradesh	1999	1431.92	1394.49	[Ratapani, Singhori, Noradehi, Kheoni, Phen, Karera, Sanjay Dubari, Bagdara]	[41.67, 45.92, 80.07, 101.47, 246.77, 309.99, 342.37, 445.17]	[139.17, 147.00, 205.70, 347.33, 107.72, 494.21, 297.18, 437.79]	[818.17, 305.50, 1306.85, 78.71, 109.61, 200.81, 808.02, 308.49]	Ratapani	41.67	139.18
29	Nagarhole	Karnataka	2000	705.89	1546.85	[Shettihalli]	[175.95]	[3.89]	[575.59]	Shettihalli	175.95	3.90

Table S1: The 29 Tiger Reserves (TRs) in our study, their candidate and selected matched Wildlife Sanctuaries (WLSs), along with TR declaration years, state located in and average annual rainfall and separating distance attributes for matching.
